# A study of the impact of AI-employee collaboration on employee innovation behavior

**DOI:** 10.3389/fpsyg.2026.1892175

**Published:** 2026-10-16

**Authors:** Yan Guanyu, Niu Chenchen

**Affiliations:** 1 School of Public Administration and Policy, Shandong University of Finance and Economics, Jinan, China; 2 School of Management, Qilu University of Technology, Jinan, China

**Keywords:** AI-employee collaboration, employee innovation behavior, self-efficacy, servant leadership, social cognitive theory

## Abstract

**Introduction:**

With the deep integration of digitalization and generative artificial intelligence, artificial intelligence has transformed from a mere technical tool into a core collaborative partner within modern workplaces and human‑resource management. As an interdependent human‑AI working mode characterized by complementary task division, joint task accomplishment, complementary decision‑making, and continuous iterative interaction between employees and AI agents, AI‑employee collaboration is conceptually distinct from simple AI adoption, instrumental AI use, and AI‑assisted technical systems. Drawing on social cognitive theory, the present study aims to investigate how AI‑employee collaboration predicts employee innovation behavior, alongside the mediating role of self‑efficacy and the moderated‑mediation boundary condition of servant leadership.

**Methods:**

Three‑wave time‑lagged questionnaire data were collected from 259 employees working in internet‑service, finance, and catering industries across Beijing and Jinan. Hierarchical regression and bootstrap approaches were adopted to test the proposed moderated‑mediation model.

**Results:**

The results show that AI‑employee collaboration exerts a significant positive predictive effect on employee innovation behavior. Self‑efficacy partially mediates the relationship between AI‑employee collaboration and employee innovation behavior. Moreover, servant leadership positively moderates the link between AI‑employee collaboration and self‑efficacy and further strengthens the indirect predictive effect of AI‑employee collaboration on employee innovation behavior through self‑efficacy.

**Discussion:**

This research enriches existing literature by providing incremental empirical evidence for the internal cognitive mechanism and contextual boundary of AI‑driven employee innovation. Theoretically, it unpacks the cognitive “black box” connecting AI‑employee collaboration and employee innovation and identifies servant leadership as a key situational contingency factor. Practically, these findings deliver concrete, actionable managerial insights for digitally transforming organizations to leverage AI tools and unlock employees’ innovative potential.

## 1 Introduction

With the explosive development of generative AI and digital intelligence technology, artificial intelligence has become a core strategic priority for enterprise transformation and upgrading (Davenport and Ronanki, 2018; Yin and Niu, 2024). AI-employee collaboration has been widely penetrated and applied across finance, Internet, catering and other service industries (Chowdhury et al., 2022; Yin et al., 2023). For instance, financial practitioners rely on intelligent algorithm systems to assist customer consulting and business decision-making; knowledge workers leverage generative AI to complete information retrieval, data computing and routine document processing (Wilson and Daugherty, 2018; Patil and Pramod, 2024).

AI is endowed with outstanding advantages in big data processing, human thinking simulation and automated decision-making. In human-workplace collaboration, AI can provide efficient, accurate and real-time auxiliary support for employees (Wang and Yao, 2022; Yin et al., 2023). As a complementary collaborative partner, AI undertakes a large number of repetitive, procedural and computational routine tasks (Frank et al., 2017; Hess and Ludwig, 2017), freeing employees from trivial affairs and enabling them to devote more time and cognitive resources to exploratory and innovative work (Susskind and Susskind, 2015; Davenport and Kirby, 2016; Mariani et al., 2023). With the gradual maturity of human-AI symbiosis paradigm, exploring how AI-employee collaboration activates employee innovation behavior has become an important theoretical and practical issue in organizational behavior and human resource management.

Although academic attention to AI-employee collaboration is rising rapidly, existing studies mainly focus on enterprise performance output, operational efficiency improvement and the dark side effect of AI application (Chowdhury et al., 2022; Makarius et al., 2020; Yin et al., 2023). Three critical research gaps remain under-explored: first, prior works pay limited attention to individual-level innovation consequences of AI-employee collaboration, and the specific influence mechanism between AI-employee collaboration and employee innovation behavior remains insufficiently explored; second, the internal cognitive path represented by self-efficacy has not been fully empirically tested; third, the contextual boundary role of servant leadership in this transmission process is also easily overlooked (Xavier and Korunka, 2025; Han et al., 2024). Unlike prior studies which often treat AI as simple technical tool or focus on AI adoption and usage behavior, this study concentrates on interdependent collaborative interaction between employees and AI agents. Building on these identified research limitations rather than framing this work as filling broad research gaps, this study draws on social cognitive theory (Bandura, 1986; Wood and Bandura, 1989) as its theoretical framework. Central to this theory is the premise that digital-work situational stimuli from AI-employee collaboration mold employees’ motivational cognitions to generate proactive work behaviors. Prior research has examined outcomes stemming from AI adoption but has not unpacked how genuine interdependent human-AI collaboration shapes cognitive beliefs to facilitate innovation. Grounded in this logic, this study constructs a moderated-mediation model: self-efficacy mediates the association between AI-employee collaboration and employee innovation behavior, whereas servant leadership moderates the first-stage path of this mediating process, yielding a classic moderated-mediation effect.

The marginal contributions of this study are reflected in two aspects: theoretically, grounded in social cognitive theory, this study identifies self-efficacy as the primary mediator, given that situational experiences of AI-employee collaboration shape innovative behaviors via individual motivational-cognitive beliefs. Meanwhile, servant leadership is explicitly framed as a moderator that alters the strength of the link between AI-employee collaboration and self-efficacy. It clarifies the cognitive mediating mechanism and leadership boundary condition of AI-employee collaboration affecting employee innovation behavior, enriches the application of social cognitive theory in human-AI collaborative scenarios, and responds to the appeal of multi-theoretical and multi-level research on AI-augmented HRM (Prikshat et al., 2021, 2023). Practically, it provides actionable management strategies for enterprises to rationally layout AI collaborative tools, cultivate employees’ innovative self-efficacy, advocate servant leadership style, and release employee innovative potential under digital transformation.

The remainder of this paper is structured as follows: literature review and research hypotheses, research design, data analysis and results, research conclusions and discussions, limitations and future research prospects.

## 2 Literature review and hypotheses

### 2.1 AI-employee collaboration and employee innovation behavior

AI-employee collaboration is defined as an interdependent human-AI working mode featured by complementary task division, joint task accomplishment, complementary decision-making, and continuous iterative interaction between employees and AI agents, where AI acts as collaborative partner rather than pure technical tool. Conceptual distinction from related constructs: AI adoption refers to organizational or individual acceptance decision toward AI technology; AI use describes simple instrumental operation behavior toward AI tools; AI-assisted work systems mainly refer to technical infrastructure. By contrast, AI-employee collaboration stresses mutual adaptation and joint performance between human agents and AI agents.

Existing literature has extensively discussed the organizational application value and workplace reshaping effect of AI technology (Haenlein and Kaplan, 2019; Daugherty et al., 2019). Compared with human individuals, AI possesses incomparable advantages in computing speed, information storage and complex data processing (Duan et al., 2019). The large-scale popularization of workplace AI is profoundly reshaping work mode, job division and organizational operation logic (Prikshat et al., 2023). AI-employee collaboration has evolved from simple tool assistance to human-machine interdependence and symbiosis, and its incremental value for individual innovation and organizational sustainable competitiveness has gradually emerged (Chowdhury et al., 2022; Yin et al., 2023; Tian and Luo, 2025).

Employee innovation behavior refers to the behavioral process that employees generate, promote and implement novel ideas in workplace practice, and form new problem-solving methods and working paradigms adapting to organizational context (Yuan and Woodman, 2010). In the era of generative AI, the threshold of employee innovation is lowered and the feasibility of innovative practice is significantly improved (Xavier and Korunka, 2025). Makarius et al. (2020) regarded AI as an important collaborative partner of employees, and pointed out that human-AI collaboration can optimize business process, improve work efficiency, and then help organizations build competitive advantages.

Social cognitive theory holds that human behavior is determined by the interactive coupling of individual cognition, behavioral intention and external environment; individual belief and motivation can significantly dominate and guide subsequent work behavior (Bandura, 1986; Qi et al., 2023). Situational input from external work environment shapes individual cognitive state, and further translates into concrete work behaviors. Under the AI-employee collaboration environment, AI undertakes massive calculation and repetitive work, greatly releasing employees’ subjective initiative and cognitive bandwidth. Positive collaborative experience with AI provides situational cues for employees. Employees have more sufficient time and energy to engage in creative thinking, scheme optimization and innovative practice. Admittedly, AI may also bring potential risks including technology dependence and deskilling effect, yet this study focuses on the positive predictive pathway under effective human-AI symbiosis conditions. Accordingly, we propose:

*Hypothesis 1:* AI-employee collaboration positively predicts employee innovation behavior.

### 2.2 The mediating role of self-efficacy

Self-efficacy is defined as individuals’ subjective confidence in their ability to accomplish specific tasks and achieve established goals (Bandura, 1977). From the perspective of social cognitive theory, work environment interaction and situational support can effectively shape and cultivate employee self-efficacy, and individual physical and mental state are closely associated with self-efficacy level (Bandura, 1986; Gong et al., 2009).

The theoretical rationale for selecting self-efficacy as the mediating variable lies in: social cognitive theory emphasizes situational input first influences individual cognitive beliefs, and then predicts behavioral outcomes (Bandura et al., 1977). AI-employee collaboration represents critical situational experience in digital workplace. Such collaborative experience cannot directly produce innovation behavior; it needs to go through individual cognitive processing. Self-efficacy, as core motivational cognitive belief, bridges external collaborative context and proactive innovation behavior. Two types of self-efficacy can be distinguished: AI-specific self-efficacy focuses on confidence specifically for operating AI technology; general self-efficacy reflects overall task-accomplishment confidence across diverse work scenarios. Positive collaborative experience working alongside AI partners may spill over and enhance employees’ general confidence for solving work tasks. It is worth noting that this spillover mechanism remains theoretically hypothesized and is not directly empirically tested in the current study.

In the AI workplace scenario, when employees perceive AI as a useful, reliable and complementary work partner, the auxiliary support provided by AI will generate positive cognitive perception and further enhance individual self-efficacy (Gursoy et al., 2019; Yin and Niu, 2024). Bandura believed that self-efficacy can affect individual behavioral choice and behavioral investment through cognitive evaluation, emotional experience, motivational drive and physiological arousal (Bandura, 1977). Recent studies further confirm that collaborative experience with generative AI can significantly shape employees’ creative self-efficacy and problem-solving self-efficacy (Patil and Pramod, 2024; Xavier and Korunka, 2025).

Higher self-efficacy will prompt employees to take the initiative to invest time and energy in innovative trial and error, dare to put forward new ideas and new methods, and thus present more proactive innovation behavior (Fang et al., 2019). Therefore, from the perspective of social cognitive theory, situational inputs such as AI-employee collaboration cannot directly trigger behavioral outcomes; they must operate through individuals’ motivational-cognitive processing. As a core agentic belief reflecting task-related confidence, self-efficacy constitutes a theoretically appropriate mediator that bridges external collaborative experiences and subsequent proactive work behaviors. Therefore, AI-employee collaboration can stimulate employee self-efficacy, and self-efficacy further promotes employee innovation behavior, playing an intermediary transmission role. We propose:

*Hypothesis 2:* Self-efficacy mediates the positive relationship between AI-employee collaboration and employee innovation behavior.

### 2.3 The moderating role of servant leadership

Servant leadership is a typical people-centered leadership style, which emphasizes listening to employee demands, caring about employee growth, and prioritizing employee development needs over self-interest (Greenleaf, 2002). Servant leadership is not inherently AI-supportive leadership; its function originates from its core attributes: autonomy support, psychological safety guarantee, learning-oriented support and tangible resource provision. In the AI-enabled workplace, these attributes help mitigate employees’ perceived uncertainty triggered by novel AI tools: servant leaders furnish practical resources and psychological reassurance for employees to engage in human-AI collaboration, buffer potential anxieties associated with adopting new intelligent technologies, and foster task-related confidence (Lin et al., 2022). Social cognitive theory emphasizes that individual cognition and behavior are embedded in the external organizational environment; leadership style, as an important situational factor, will interact with individual cognitive perception and jointly affect behavioral output (Bandura, 1986; Qi et al., 2023).

Servant leaders are willing to provide resource support and emotional care, establish high-quality communication connection with employees, fully recognize employees’ ability characteristics and development goals, and actively empower employees to realize personal potential, thereby helping enhance employee confidence and work engagement (Greenleaf, 2002; Ye et al., 2022). When employees collaborate with AI, such leader-provided resources and psychological security enable them to better capitalize on the benefits of AI assistance. Consequently, the positive influence of AI-employee collaboration on self-efficacy becomes amplified under high servant leadership. A large number of studies have verified that servant leadership has a significant predictive effect on employee attitude, psychological state and proactive behavior (Dierendonck et al., 2014; Newman et al., 2017; Chen et al., 2015).

Servant leaders offer psychological safety for employees to try new work modes including AI-enabled collaboration, grant autonomy for employees to arrange human-AI task allocation, and provide learning resources to reduce uncertainty brought by new AI tools. Verbal encouragement, behavioral support and growth empowerment from servant leadership are important antecedents of employee self-efficacy (Zhang et al., 2010). When servant leadership is at a high level, leaders create safe atmosphere for employees’ AI workplace practice, provide resource guarantee and psychological encouragement, so as to amplify the positive impact of AI-employee collaboration on self-efficacy. When servant leadership is at a low level, leaders lack autonomy support, psychological safety supply and resource input for employees’ new-mode work practice, weakening the positive contribution of AI collaboration to self-efficacy. Therefore:

*Hypothesis 3:* Servant leadership positively moderates the relationship between AI-employee collaboration and self-efficacy, i.e., the higher the level of servant leadership, the stronger the positive association of AI-employee collaboration with self-efficacy.

Furthermore, this study proposes a moderated mediation model. Servant leadership can further regulate the mediating strength of self-efficacy in the relationship between AI-employee collaboration and employee innovation behavior. Under high servant leadership, the indirect promoting effect of AI-employee collaboration on innovation behavior via self-efficacy is stronger; under low servant leadership, the indirect effect is relatively weaker. Thus:

*Hypothesis 4:* Servant leadership moderates the mediating effect of self-efficacy between AI-employee collaboration and employee innovation behavior. Specifically, when the level of servant leadership is higher, the indirect effect is stronger; when lower, the indirect effect is weaker.

## 3 Research design

### 3.1 Sample and data collection

Participants were recruited via purposive-convenience sampling. We targeted full-time employees from internet services, catering, and financial enterprises located in Beijing and Jinan. These three industries were chosen because AI collaborative tools have been widely integrated into daily workflows for customer service, data analysis, decision-making support, and workflow optimization, offering a realistic empirical context for studying AI-employee collaboration (Figure 1).

**Figure 1:**
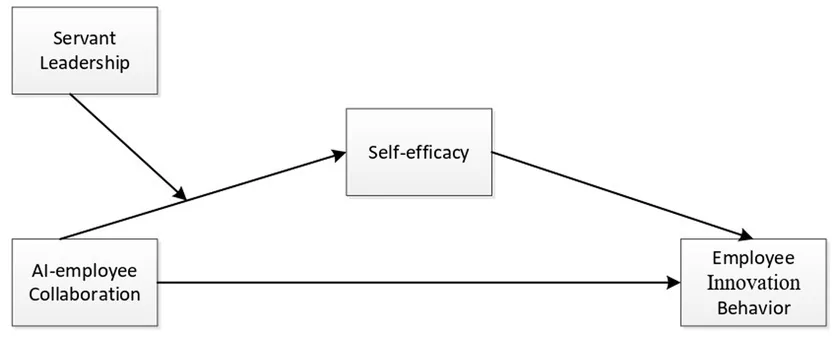
Research model.

To ensure sample validity, we embedded screening items in the Time-1 questionnaire: participants reporting zero regular work-related AI usage were excluded from the final dataset. It should be noted that this screening only eliminates complete absence of workplace-AI experience, while allowing heterogeneity in AI-usage intensity among retained respondents. We further collected descriptive information concerning respondents’ AI-tool types, frequency of workplace AI use, AI-relevant work experience, and primary task scenarios supported by AI. The final matched valid sample comprised 259 full-time employees, including 128 males (49.3%) and 131 females (50.7%). Participants’ average age was 28.35 years (SD = 4.52), and their average organizational tenure was 4.16 years (SD = 2.38). In terms of AI-usage profiles: 82.6% of participants utilized generative AI, algorithm-driven decision-support systems, or service-oriented AI tools at high frequency in their jobs; the remaining 17.4% of valid participants still had regular work-related AI exposure but with relatively lower usage intensity, with weekly AI-related working hours ranging from 3 to 15 h. AI-supported work tasks spanned information retrieval, data computation, customer service, document drafting, and auxiliary decision-making.

This study employed a three-wave time-lagged research design with a two-week interval between each wave. Questionnaires were disseminated through a professional domestic online survey platform. At Time 1, participants completed measures of demographic information, AI-employee collaboration, and servant leadership. At Time 2, respondents reported their self-efficacy. At Time 3, participants assessed their employee innovation behavior. In total, 300 questionnaires were distributed at Time 1, yielding 279 returned surveys; 268 valid responses were collected at Time 2, and 259 matched valid responses were retained at Time 3, corresponding to an effective response rate of 86.3%. A small cash incentive of RMB 3 was provided to participants upon completion of each survey wave to boost response rates and data quality.

### 3.2 Measurement

All variables were measured using mature international published scales. To adapt to the Chinese organizational context, the standard translation-back translation procedure was adopted: the original English scale was first translated into Chinese, then back-translated into English by professional independent translators. Items were revised item by item to ensure semantic consistency and cultural adaptability. All items adopted a 5-point Likert scale (1 = strongly disagree, 5 = strongly agree).

#### 3.2.1 AI-employee collaboration

At present, no well-established, off-the-shelf validated scale specifically captures interdependent AI-employee collaboration featuring complementary task division, joint judgment, and iterative human-AI interaction for general organizational employees. Existing published instruments predominantly evaluate the degree of AI involvement in work workflows, yet they fail to reflect the reciprocal interdependence between employees and AI agents (Kong et al., 2023). Given this research gap, four items in this study are theoretically derived based on conceptual dimensions from seminal human-AI collaboration literature (Seeber et al., 2020; Kong et al., 2023), instead of being directly adapted from any previously-published validated measurement scale.

AI-employee collaboration refers to an interdependent human-AI working mode characterized by complementary task division, joint task accomplishment, complementary decision-making, and continuous iterative interaction between employees and AI agents, in which AI acts as a collaborative teammate rather than merely an instrumental tool. The four items are as follows: (1) I maintain independent thinking when using AI tools for work; (2) I can cooperate well with AI tools to complete work tasks; (3) I will make joint judgment with AI output results when dealing with work affairs; and (4) My work and AI output form complementary relations. Responses were collected on a 5-point Likert scale (1 = strongly disagree to 5 = strongly agree). Cronbach’s α = 0.87 and CFA results demonstrate acceptable item-level psychometric performance. Nevertheless, It should be noted that reliability and confirmatory factor-analysis results do not replace formal expert-panel assessment of content validity. As such, further systematic examination, ideally within dedicated scale-development work, would be valuable to consolidate the content validity of this construct.

#### 3.2.2 Servant leadership

This study employed a scale developed by Liden et al. (2014), comprising seven items. Employees self-assessed the perceived level of servant leadership on a scale from 1 to 5. Example item: “My leader provides me with opportunities to develop my skills.” Cronbach’s α = 0.91.

#### 3.2.3 Self-efficacy

This study utilized the self-efficacy measurement scale developed by Schwarzer and Jerusalem (1995). We conducted preliminary item analysis according to factor loading results, and selected first six items with high factor loadings. These reserved items focus on general task confidence and problem-solving belief, matching our theoretical mechanism. Participants rated their self-efficacy on a scale of 1 to 5. Example item: “If I exert the necessary effort, I am confident I can solve most problems.” Cronbach’s α = 0.89. We note that this is general self-efficacy scale rather than AI-specific self-efficacy scale. The theoretical rationale for using general self-efficacy: positive collaborative experience with AI spills over and shapes employees’ overall task confidence across work contexts. Nevertheless, we admit AI-specific self-efficacy scale would be more ideal for AI-collaboration research, and we mark this as study limitation. We acknowledge that AI-specific self-efficacy would constitute a more direct matching measure for AI-collaboration research. The spillover assumption that AI-collaboration experience shapes general self-efficacy is a theoretical conjecture without direct empirical test in this paper. Future studies are encouraged to simultaneously collect AI-specific self-efficacy and general self-efficacy data to verify this spillover logic.

#### 3.2.4 Employee innovation behavior

This study employed a scale developed by Parker and Collins (2008) consisting of three items, used for self-assessment of the frequency of innovation behavior, rated on a scale from 1 (“never”) to 5 (“very frequently”). Example item: “I frequently propose creative ideas and suggestions.” Cronbach’s α = 0.88.

#### 3.2.5 Control variables

Drawing from prior innovation research, this study controlled for gender, age, and work experience (Gong et al., 2013; Lin et al., 2017; Madjar et al., 2002).

## 4 Data analysis and results

### 4.1 Common method variance test

This study adopted three-stage time-lagged design to alleviate common method variance. Harman single-factor test was conducted via SPSS 23. The variance explained by the unrotated first principal component was 26.45%, far lower than the 50% critical standard, indicating that common method variance was not a serious threat to the research conclusion.

### 4.2 Confirmatory factor analysis

Mplus 7.0 was used for confirmatory factor analysis to test discriminant validity. The four-factor model (AI-employee collaboration, servant leadership, self-efficacy, employee innovation behavior) fitted the data best: χ^2^/df = 2.38, RMSEA = 0.064, CFI = 0.912, GFI = 0.927, showing good discriminant validity of all variables (Table 1).

**Table 1:** Confirmatory factor analysis results.

Models	χ2/df	RMSEA	CFI	GFI
Four-factor model	2.38	0.064	0.912	0.927
Three-factor model	3.31	0.083	0.857	0.882
Two-factor mode	4.26	0.097	0.793	0.801
One-factor model	4.87	0.124	0.654	0.712

### 4.3 Descriptive statistics

Correlation results showed that AI-employee collaboration was positively correlated with self-efficacy (*r* = 0.426, *p* < 0.01); AI-employee collaboration was positively correlated with employee innovation behavior (*r* = 0.245, *p* < 0.01); self-efficacy was positively correlated with employee innovation behavior (*r* = 0.432, *p* < 0.01). The correlation direction and significance initially supported the research hypotheses (Table 2).

**Table 2:** Results of descriptive statistics.

Variable	AI-employee collaboration	Self-efficacy	Servant leadership	Employee innovation behavior
AI-employee collaboration	1			
Self-efficacy	0.426**	1		
Servant leadership	0.315**	0.386**	1	
Employee innovation behavior	0.245**	0.432**	0.329**	1

** Means *p* < 0.01.

### 4.4 Hypotheses testing

#### 4.4.1 Relationship between AI-employee collaboration and employee innovation behavior

Hierarchical regression analysis (Table 3) showed that after controlling for demographics, AI-employee collaboration positively predicted employee innovation behavior (*β* = 0.316, *p* < 0.01), 95% CI [0.142, 0.571]. Hypothesis 1 was supported.

**Table 3:** Results of stratified regression test.

Variable	Employee innovation behavior			Self-efficacy		
	Model 1	Model 2	Model 3	Model 4	Model 5	Model 6
Gender	0.135^*^	0.127^*^	0.149^*^	0.019	0.013	0.025
Age	0.068	0.084	0.075	0.079	0.091	0.084
Working years	0.168	0.154	0.127	0.136^*^	0.143^*^	0.131^*^
AI-employee collaboration		0.316^**^	0.297^**^		0.284^**^	0.235^**^
Self-efficacy			0.216^**^			
Servant leadership						0.125^*^
AI-employee collaboration × servant leadership						0.226^**^

* Means *p* < 0.05; ** means *p* < 0.01.

#### 4.4.2 Mediating effect of self-efficacy

Following Wen et al. (2004, 2022), we tested mediation. First, AI-employee collaboration significantly predicted employee innovation behavior (Model 2: *β* = 0.316, *p* < 0.01). Second, AI-employee collaboration significantly predicted self-efficacy (Model 5: *β* = 0.284, *p* < 0.01), 95% CI [0.129, 0.432]. Third, when both AI-employee collaboration and self-efficacy were entered, self-efficacy significantly predicted innovation behavior (Model 3: *β* = 0.216, *p* < 0.01), and the direct effect of AI-employee collaboration remained significant but reduced (*β* = 0.297, *p* < 0.01). We further supplemented bootstrap test results: indirect effect value = 0.061, 95% bootstrap CI [0.023, 0.107], mediation effect size = 0.061, and self-efficacy accounts for 16.23% of total effect. Thus, self-efficacy partially mediated the relationship. Hypothesis 2 was supported.

#### 4.4.3 Moderating effect of servant leadership

AI-employee collaboration and servant leadership were mean-centered. Model 6 (Table 3) showed that the interaction term significantly predicted self-efficacy (*β* = 0.125, *p* < 0.01), 95% CI [0.124, 0.386], indicating moderation. Simple slope analysis revealed that when servant leadership was high (+1 SD), the effect of AI-employee collaboration on self-efficacy was *β* = 0.412, *p* < 0.01; when low (−1 SD), *β* = 0.156, *p* < 0.05 (Figure 2). Hypothesis 3 was supported.

**Figure 2:**
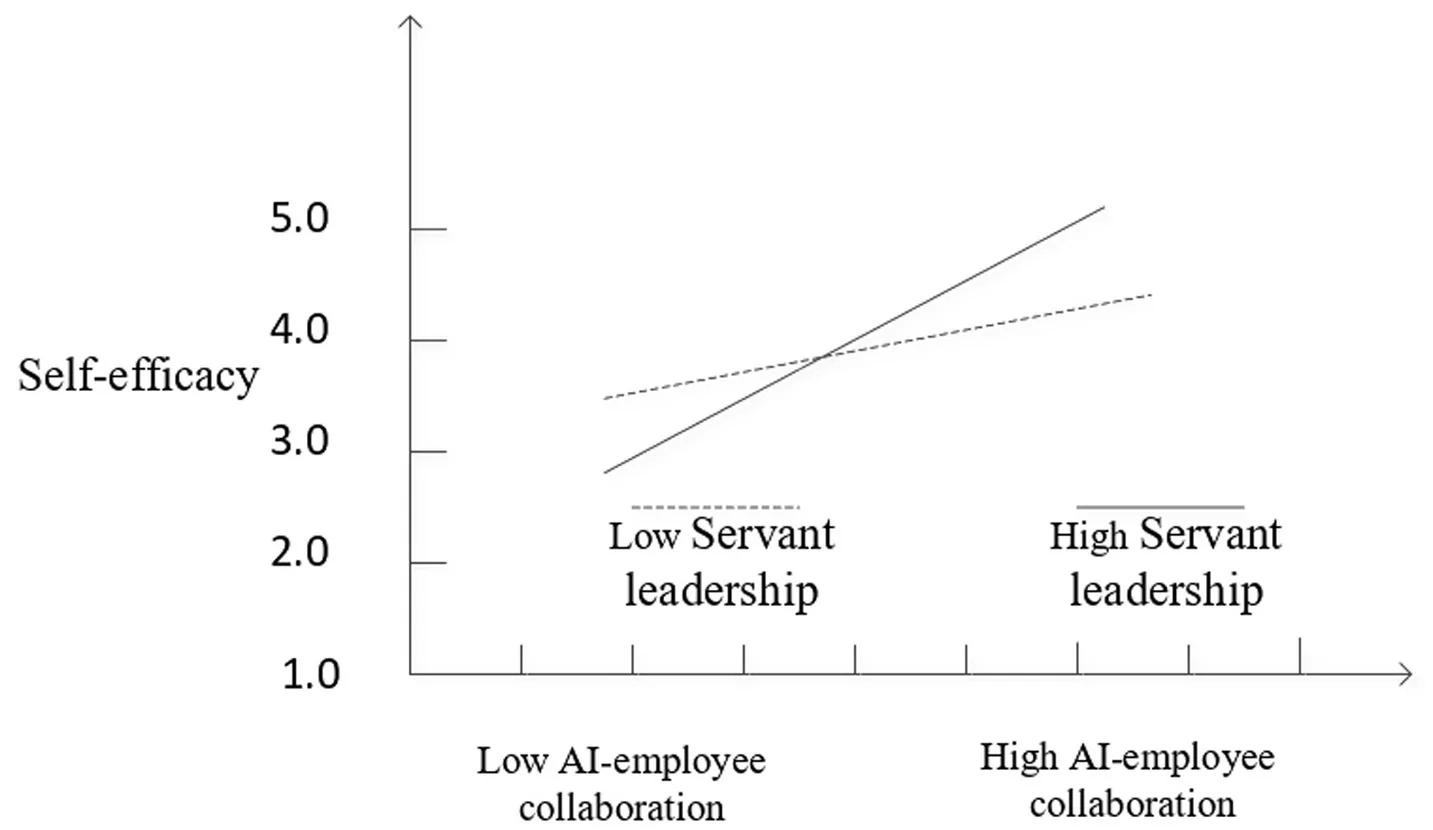
Moderating role of servant leadership in the relationship between AI-employee collaboration and self-efficacy.

#### 4.4.4 Moderated mediation effect

We followed Wen and Ye (2014) and additionally computed the index of moderated mediation using PROCESS (Model 7; Hayes, 2018). The index was 0.078 (SE = 0.029, 95% CI [0.024, 0.139]), indicating that the indirect effect varies significantly with servant leadership. Consistent with the three-step regression results (Table 4), Hypothesis 4 was supported: servant leadership moderated the mediating effect, such that the indirect effect was stronger under high servant leadership.

**Table 4:** The results of moderated mediation effect.

Variable	Employee innovation behavior		Self-efficacy
	Model 1	Model 3	Model 2
Gender	0.125^*^	0.146^*^	0.025
Age	0.083	0.073	0.084
Working years	0.152	0.124	0.131^*^
AI-employee collaboration	0.273^**^	0.242^**^	0.235^**^
Self-efficacy		0.209^**^	
Servant leadership	0.196^*^	0.123^*^	0.125^*^
AI-employee collaboration × servant leadership	0.243^**^	0.221^**^	0.226^**^

* Means *p* < 0.05; ** means *p* < 0.01.

## 5 Conclusions and discussion of the study

### 5.1 Research findings

Based on social cognitive theory, this study systematically explored the influence mechanism and boundary condition of AI-employee collaboration on employee innovation behavior, and all four hypotheses were statistically verified.

First, AI-employee collaboration can significantly promote employee innovation behavior. This empirical result supports the core proposition of social cognitive theory that favorable situational conditions can facilitate individuals’ proactive work behaviors (Bandura, 1986). Beyond merely confirming that AI frees employees’ time and cognitive resources as documented in prior research (Davenport and Kirby, 2016), our finding further validates that the interdependent collaborative relationship between human and AI agents—rather than simple tool usage—is an important driver of employee innovation. This result complements the work of Chowdhury et al. (2022) by shifting research focus from firm-level performance outcomes to individual-level innovative behavioral outcomes. Meanwhile, this result does not contradict existing “double-edged sword” findings reported by Yin et al. (2023): our evidence reflects the positive pathway under effective human-AI symbiosis, while negative consequences such as technology dependence may emerge under poor AI implementation conditions.

Second, self-efficacy partially mediates the relationship between AI-employee collaboration and employee innovation behavior; AI collaboration enhances employees’ task confidence and problem-solving belief, thereby stimulating innovation behavior. This finding supports the core cognitive mechanism of social cognitive theory: external situational experiences are transformed into work behaviors through individual cognitive-motivational beliefs (Bandura, 1977). It responds to the research call from Xavier and Korunka (2025) to uncover the cognitive “black box” linking human-AI interaction and employee innovation. Different from prior studies that mostly focused on creative-specific self-efficacy, we treat this spillover as theoretical conjecture rather than firmly established empirical conclusion, which in turn fuels innovative behaviors. This offers a supplementary cognitive explanation for AI-driven employee innovation.

Third, servant leadership positively moderates the impact of AI-employee collaboration on self-efficacy; high servant leadership can amplify the positive cognitive gain brought by human-AI collaboration. This result extends the application scope of social cognitive theory by verifying the interactive effect of situational leadership factor and digital work context on individual cognition (Bandura, 1986). Consistent with classical servant-leadership research (Liden et al., 2014; Newman et al., 2017), our study demonstrates that servant leadership exerts its influence via autonomy support, psychological safety and resource supply, instead of simply advocating for AI technology. It further explains why identical AI-collaboration conditions may generate heterogeneous cognitive responses among employees under different leadership contexts.

Fourth, servant leadership significantly moderates the mediating effect of self-efficacy, forming an obvious moderated mediation effect. This moderated-mediation result integrates digital workplace context with servant-leadership theory. It clarifies the conditional boundary: the psychological benefit brought by AI-employee collaboration is not unconditional. The positive indirect pathway from AI-employee collaboration to innovation via self-efficacy is more pronounced when organizations have high-quality servant leadership. This conclusion adds new contingency evidence for AI-augmented human resource management research (Prikshat et al., 2023), and provides a cross-level theoretical explanation for why some firms fail to harvest innovation benefits even after deploying advanced AI collaborative tools.

### 5.2 Theoretical implications

First, this study expands the application boundary of social cognitive theory to human-AI collaborative workplace scenarios. Different from prior AI-related studies which mainly focus on resource-release logic brought by AI tools, this study highlights that AI-employee collaboration as situational experience shapes individual cognitive belief, which further transfers to work behaviors. This study responds to the call for applying social cognitive theory in human-AI symbiosis context (Xavier and Korunka, 2025). Prior scholarship has documented how instrumental AI assistance influences employee innovation and creative-specific self-efficacy. Most prior work centers on one-way AI-tool usage: employees receive outputs from AI systems without deep interdependent joint-task interaction. By contrast, this study highlights interdependent AI-employee collaboration characterized by complementary task division, joint judgment, and continuous iterative human-AI interaction. Such interactive collaborative experience creates distinct situational inputs, which may generate cognitive consequences different from simple instrumental AI adoption or passive AI-assistance. This constitutes our key incremental theoretical contribution. Second, this study reveals the internal cognitive transmission path of self-efficacy, opens the “black box” between AI-employee collaboration and employee innovation behavior. Complementing prior AI innovation research that emphasizes output variables, our work proves that general self-efficacy serves as an important psychological bridge linking AI-employee collaboration and innovation behavior. We compare our findings with Yin et al. (2023)’s double-edged-effect research: our study focuses on positive predictive pathway under effective human-AI symbiosis, without denying potential negative effects of AI. Third, this study introduces servant leadership as the situational boundary, clarifies when AI collaboration can better enhance self-efficacy and innovation behavior. We demonstrate servant leadership works through autonomy support, psychological safety and resource provision rather than simple AI-advocacy.

### 5.3 Practical implications

First, enterprises should actively layout AI collaborative tools, optimize human-machine task division, let AI undertake repetitive work, and reserve cognitive space for employee innovation. When designing human-AI task allocation, managers need to avoid over-reliance on AI and prevent excessive task substitution that may trigger employee job insecurity. Notwithstanding the innovation-promoting benefits of AI-employee collaboration, organizations should recognize practical challenges accompanying AI deployment, including employee resistance toward new intelligent tools, algorithmic ethical risks, data privacy concerns, and insufficient AI transparency, all of which may undermine the expected gains from human-AI collaboration. Second, organizations should carry out AI skill training and achievement feedback mechanism to continuously enhance employees’ work self-efficacy and innovative confidence. Targeted training programs should also cover psychological adaptation guidance to mitigate employee resistance, reduce fear of being replaced by AI, and help employees build positive mindsets toward human-AI cooperation. Meanwhile, firms need to establish supporting rules for AI application: formulate internal norms for data collection and usage, strengthen data privacy protection for employee and client information, and improve the transparency of AI algorithm outputs. For instance, organizations can clarify the scope of AI decision-making, ensure employees understand the generation logic and limitation of AI-produced outputs, and avoid blind trust in algorithmic results. Third, managers should cultivate and practice servant leadership, attach importance to employee growth demand, provide resource support and emotional encouragement in AI transformation. Servant leaders should create open communication channels for employees to voice worries about AI-related risks and ethical dilemmas, rather than only promoting the advantages brought by AI tools. By addressing these practical barriers, organizations can mitigate the dark-side risks of AI application and release the innovation driving value of AI-employee collaboration to the maximum extent.

## 6 Limitations and future research

This study has several limitations, and we further elaborate how these limitations influence research interpretation and generalizability. First, despite using a time-lag design, the three-time-point design with short intervals may still be affected by common method variance. This may inflate correlation among variables and restrict robust causal inference. Future research should use multi-stage, multi-source designs. Second, we used convenience-purposive sampling; the sample cannot fully represent all types of employees. Mixed methods could improve robustness. Third, the sample was limited to China and three industries. Even though these industries own AI-collaboration scenarios, cross-industry AI application heterogeneity exists, which may restrict cross-sector generalizability. Cross-country and cross-industry replication is needed. Fourth, we only used social cognitive theory; future studies could apply social information processing theory or attribution theory. Fifth, given the predictive non-experimental design, the reported predictive relationships should not be interpreted as strict causal relationships. Longitudinal or experimental designs are needed to establish causality. Sixth, we adopted shortened general self-efficacy scale instead of AI-specific self-efficacy measurement, which cannot fully capture cognition specifically targeting AI technology, possibly attenuating effect estimation for AI-related psychological path. Seventh, our four-item AI-employee collaboration measure is self-adapted. Although acceptable reliability and CFA indices are obtained, content validity cannot be fully guaranteed, and dedicated scale development is needed in future research. Eighth, we employ general self-efficacy instead of AI-specific self-efficacy. The proposed spillover mechanism, whereby AI-collaboration experience enhances general self-efficacy, remains a theoretical conjecture and is not directly tested in the present study.

For future research directions, more specific suggestions are proposed. First, future research can distinguish different types of AI, such as generative AI, conversational AI, and decision-support AI, and compare their divergent influence on employee cognition and innovation behavior. Second, scholars can introduce additional psychological mechanisms such as trust in AI, AI literacy, and psychological safety to enrich the psychological path of AI-employee collaboration. Third, future works can explore AI governance issues including algorithm ethics, transparency, fairness, and data privacy in human-AI collaboration context. Fourth, researchers can adopt AI-specific self-efficacy or creative self-efficacy scales to replicate our mediating mechanism. Fifth, cross-industry comparative research is encouraged to explore industry heterogeneity for AI-employee collaboration effects.

## Data Availability

The datasets presented in this study can be found in online repositories. The names of the repository/repositories and accession number(s) can be found in the article/supplementary material.
